# Analysis of the drainage effect of different incisions for high complex anal fistula based on FLUENT hydrodynamic simulation

**DOI:** 10.3389/fsurg.2022.974341

**Published:** 2022-08-12

**Authors:** Jiamin Zhang, Xiang Li, Jiaze Ma, Peng Chen, Wanli Li, Junjie Hu, Xiaoliu Li, Yile Chen, Kang Ding

**Affiliations:** ^1^ Nanjing University of Chinese Medicine, Graduate School of Nanjing University of Chinese Medicine, Nanjing, China; ^2^ Colorectal Disease Center of Nanjing Hospital of Chinese Medicine, Nanjing University of Chinese Medicine, Nanjing, China; ^3^ Anorectal surgery Department of Suqian Hospital of Chinese Medicine, Nanjing University of Chinese Medicine, Suqian, China

**Keywords:** high complex anal fistula, numerical simulations, incision shape, drainage effect, biomechanics

## Abstract

**Purpose:**

The biomechanical characteristics of the trauma size and postoperative drainage of different incisions for high complex anal fistula surgery were compared by numerical simulation analysis to provide a theoretical basis for the clinical selection of minimally invasive incisions for surgery.

**Methods:**

Using FLUENT finite element software, a typical incision finite element model was established to obtain incision areas, and the total mass outlet flow within 200 s was calculated to evaluate the drainage effect of each incision.

**Results:**

The incisions with the largest to smallest areas were the curved, spindle, and curved plus extended groove incision, indicating that the curved plus extended groove incision caused the least damage to the perianal skin muscles. Conversely, the incisions with the largest to smallest total outlet flow were as follows: curved plus extended groove, spindle, curved, and straight incision, suggesting that the curved plus extended groove model had the best diversion effect, and the curved incision had better diversion effect than that of the straight incision.

**Conclusion:**

The curved plus extended groove surgical incision had the smallest incision area, minimized damage to the perianal skin and muscle tissue, conformed to the concept of minimally invasive surgery, ensured adequate drainage of exudate, maintained the normal growth of granulation tissue on the wound surface, preserved the original form of the anus, and thus better protected the function of the anus. This improved the quality of life of patients requiring high complex anal fistulas.

## Introduction

Complex anal fistulas can involve the external sphincter, supra-sphincter, trans-sphincter involving >30% of the external anal sphincter, and anterior trans-perineal complex in female patients; be horseshoe in nature; as well as be in the form of fistulas in combination with inflammatory bowel disease, radiation enteritis, malignant tumors, anal incompetence, and chronic diarrhea (1). In current clinical practice for surgical treatment of a high-grade complex anal fistula, incisions that are too small negatively affect drainage in the preliminary treatment, resulting in the failure to repeat the pus outflow. In contrast, an overly large incision affects the integrity and prognostic function of the anus. Different incision shapes distribute and can alter treatment outcomes, and there is no uniform clinical standard for incision settings, nor is there a quantitative standardized operational study. Thanks to the development of finite element commercial software and cross-disciplinary applications, many scholars (2–5) have used finite element numerical software to simulate the mechanical motion characteristics of human tissues under varying conditions and achieved better results using the software application combined with clinical practice. In this study, we established several typical finite element models of incision based on FLUENT finite element software to simulate and analyze the drainage effects of different postoperative incisions in patients with anal fistulas.

## Materials and methods

### Materials

Three-dimensional ultrasonography was performed on ten patients with high complex anal fistulas who received different incision shapes to clarify the fistula pathway. While the postoperative incision shapes varied, all approximated a conical shape with different base surfaces. Magnetic resonance imaging (MRI) scans were also performed postoperatively to obtain the structure of the internal and external anal sphincter, pelvic floor anal levator, anal margin tissues, and incision, in order to precisely locate the position, size, and location of the apex of the incision and the curved shape of the incision surface. Data from MRI scans were used as the basis for geometric modeling of the postoperative wound drainage mechanics model; the postoperative incisions were thus approximated as cone models with different base shapes, which were classified as either a spindle, curved, or curved plus extended groove incisions according to the shape of the base. The primary research equipment and software are displayed in Tables 1, 2, respectively.

**Table 1:** Manufacturer and model of the main equipment.

Equipment	Manufacturer	Model
Digital Viscometer	Shanghai Jingtian Electronic Instrument Company with Limited Liability	DV-1
Digital display thermostatic water bath	Jiangsu Jinyi Instrument Technology Company with Limited Liability	HH-S1

**Table 2:** Manufacturer and model of the main software.

Software	Manufacturer	Model
SolidWorks	Dassault Systems	2021
ANSYS mesh	ANSYS	2020 R1
FLUENT	ANSYS	2020 R1

### Methods

#### Incision dimensions

The length (longest distance of the incision), width (widest distance perpendicular to the length), and depth (distance from the tip of the pus cavity to the incision margin) of the incisions were measured using a nano-soft ruler in 70 postoperative anal fistula patients at Nanjing Hospital of Traditional Chinese Medicine (Table 3).

**Table 3:** Range of incision size after anal fistula operation.

Incision	Distance (mm)
Length	41.39 ± 18.34
Width	24.40 ± 15.69
Depth	32.84 ± 14.90

#### Postoperative weight of secretion

Gauze was obtained postoperatively from 30 patients with anal fistulas, and the difference in gauze weight before and after dressing change was measured to derive the weight of wound exudate from the patient's postoperative day to six days after surgery. The average amount of secretion on the postoperative day was 30 g.

#### Parameters of non-Newtonian fluid model

Experimental data on the viscosity of liquids at a temperature of 34 °C were measured using a digital viscometer at a room temperature of 27 °C (Table 4).

**Table 4:** Viscosity test data at a temperature of 34 °C.

Experimental instruments	Rotational Speed (rpm)	Viscosity (mPa.s)	Shear rate (1/s)
Digital display thermostatic water bath: 34 °C (Digital Viscometer: 34.4 °C)	40.0	91.8	13.6
	30.0	144.1	10.2
	20.0	170.8	6.8
	10.0	280.1	3.4
	5.0	509.8	1.7
	4.0	836.6	1.4

**Figure 1:**
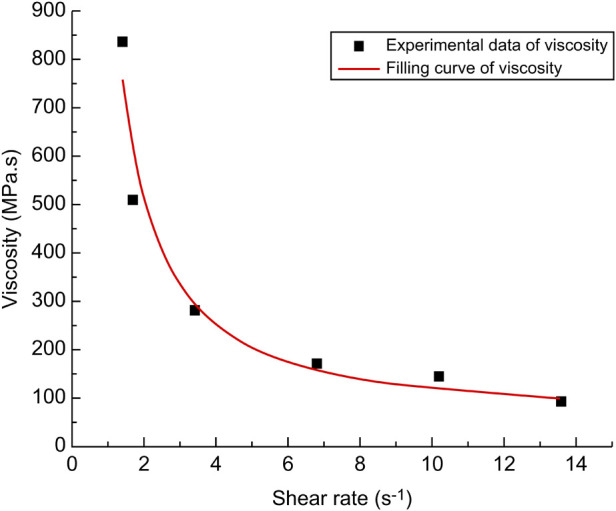
A plot of the viscosity fitting characteristics at a temperature of 34 °C.


The origin was used to fit the data to obtain a plot of the viscosity fitting characteristics (Figure 1), which led to the calculation of the four parameters required for the non-Newtonian fluid model Carreau model (6). The parameters were: $\mu_{\infty }$ (infinite shear rate viscosity)  ≈ 52.8 mpa·s, $\mu_{0}$ (zero shear rate viscosity) ≈ 1,702,216.5 mpa·s, *λ* (unit time parameters) ≈ 548.4, *n* (dimensionless parameters) = −0.2. The viscosity model characterized the relationship between fluid viscosity and shear rate, as shown in the following Equation (1).

(1) $$\mu =\mu_{\infty \;}+\;\left(\mu_{0}-\mu_{\infty }\right){\left[1+{\left(\lambda \gamma \right)}^{2}\right]}^{\frac{{}_{n-1}}{{}_{2}}}$$

#### Building the model

According to the different forms of incisions now used in the clinic, the ideal models of six typical incisions were built by SolidWorks. These included the straight model of the spindle incision, straight model of the curved incision, straight model of the curved plus extended groove incision, curved model of the spindle incision, curved model of the curved incision, and curved model of the curved plus extended groove incision. The geometric model was meshed in the Ansys mesh module, imported into FLUENT, and a uniform inlet boundary condition and constant outlet pressure were set to simulate the drainage process of different incision models.

**Figure 2:**
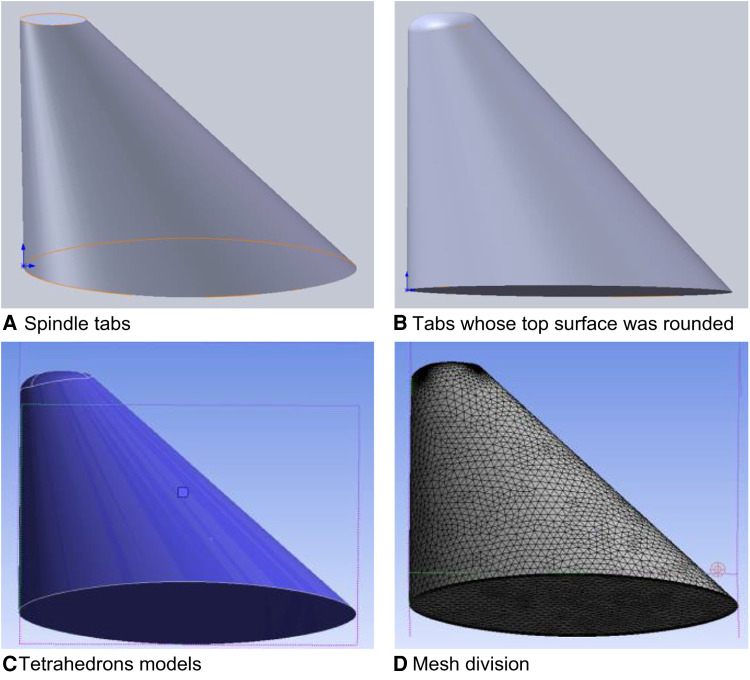
Schematic diagram of the straight incision model with spindle shape.

Taking the straight model of spindle incision as an example, two datum planes were first established in commercial software (SolidWorks 2017), where the vertical distance between the two datum planes was 32.84 mm. Second, the spindle shapes were sketched on the two datum planes in order; then, the corresponding end points were connected, and the guide lines were established. Third, by using the two spindle shapes as contours, the tab/substrate was placed and the top surface was rounded, where the radius of the rounded corner was 3 mm (Figure 2). Finally, the model was imported into Ansys to calculate the inlet/outlet area, and the tetrahedrons tetrahedral cell in the Ansys mesh module was selected to mesh the geometric model with a cell size of 0.001 mm. In this process, the boundary layer was densified so that the maximum number of layers was five; the transition ratio was 0.272; and the growth rate was 1.2. Similarly, other incision models were established, and the relevant model information is shown in Table 5.

**Table 5:** Model information.

Names	Sizes (mm)	Mesh size	Number of nodes (pcs)	Number of units (pcs)
Straight model of spindle incision	41.39 × 24.40 × 32.84	1	150,727	524,920
Curved model of spindle incision	41.39 × 24.40 × 32.84	1	126,987	434,574
Straight model of curved incision	41.39 × 24.40 × 32.84	1	179,381	89,266
Curved model of curved incision	41.39 × 24.40 × 32.84	1	119,933	91,267
Straight model of curved plus Extended groove incision	41.39 × 24.40 × 32.84	1	96,103	533,557
Curved model of curved plus Extended groove incision.	41.39 × 24.40 × 32.84	1	43,511	334,633

### Observation index and calculations

#### Different incision areas

Outlet area was calculated by importing each model into Ansys.

#### Analysis of mass flow inlet

The total flow rate of tissue fluid *Q* for one day was set at 30 g, and the density as *ρ *= 1 g/m^3^ (liquid density was the same as water density). Therefore, the volume of tissue fluid was set as 30 ml. The straight model of spindle incision was selected as the standard model for 30 g/day of tissue fluid, and the model inlet area *S* was read, from which the mass flow rate *v*_m_ was obtained with the following Equation (2).


(2) $$v_{m}=\;\frac{Q}{t}=\frac{30\times 10^{-3\;}\;\mathrm{kg}}{24\times 60\times 60s}=3.472\times 10^{-7}\;kg/s$$

The inlet velocity flow rate *v* was obtained after bringing in the inlet area of different models. Taking the straight model of spindle incision as an example:

(3) $$v\;=\frac{Q}{\rho St}=1.5\times 10^{-7\;\;}\;m/s$$

#### Numerical calculation theory of the volume of fluid (VOF) two-phase flow

The VOF relies on two non-interpenetrating fluids, and the volume fraction of each phase in the model can be calculated (7); that is, the sum of the volume fractions of all phases in each control body is one (8). Using FLUENT finite element software, a typical incision finite element model was established. Since the secretion process of incision pus and secretion is the inflow of pus and secretion into the air, which involves the interaction between liquid and gas, the VOF two-phase flow model was used, with air set as the main term and liquid set as the secondary term. The air-liquid mixture flow existed in the system.

**Figure 3:**
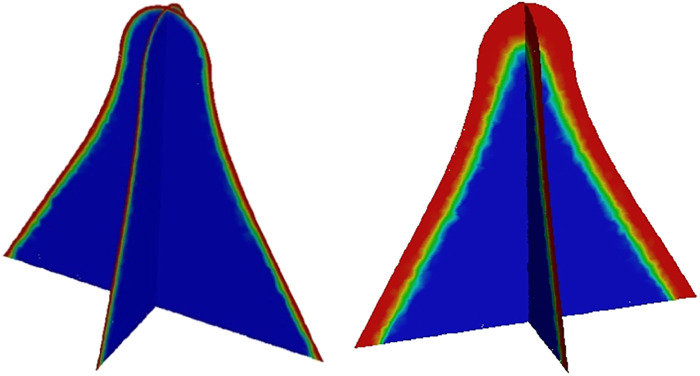
Volume fraction of secretion at different moments in the longitudinal and transverse sections of the spindle incision.

The volume fraction at different moments of the spindle incision is illustrated, where the red area is the postoperative residual pus and secretion, and the blue part is air. It can be clearly seen that there is a clear separation interface between secretion and air, as well as the flow state of secretion (Figure 3).

Numerical simulation of wound drainage effects was based on the Navier-Stokes (N-S) equation theory (9). The outlet flow rate was monitored, and an outlet mass flow rate monitoring plot was obtained, yielding a 100-fold increase in velocity over 200 s. Considering f(t) to be the outlet mass flow rate as a function of time t, the outlet mass flow curve was wavy and subject to slight reflux due to the viscous buildup effect of tissue fluid. By integrating f(t) through the numerical integration method, the total outlet flow *Q* in 200 s for different incisions could be expressed as the integral Equation (4).


(4) $$Q=\int_{0}^{200}\;f\;(t)dt$$

### Ethics approval and consent to participate

All methods were carried out in accordance with relevant guidelines and regulations. All experimental protocols were approved by the ethics committee of Nanjing Hospital of Chinese Medicine Affiliated to Nanjing University of Chinese Medicine (Nanjing, China). Written informed consent was obtained from all study subjects or their guardians.

## Results

The outlet area of different incisions is shown in Table 6.

**Table 6:** Size and inlet/outlet area of models.

Names	Size (mm)	Inlet area (m^2^)	Outlet area (m^2^)
Straight model of spindle incision	41.39 × 24.40 × 32.84	0.00255830231	0.00085895
Curved model of spindle incision	41.39 × 24.40 × 32.84	0.0022617451	0.00085895
Straight model of curved incision	41.39 × 24.40 × 32.84	0.00471548585	0.0010334
Curved model of curved incision	41.39 × 24.40 × 32.84	0.00442804	0.00102991
Straight model of curved plus extended groove incision	41.39 × 24.40 × 32.84	0.00421396	0.00059484
Curved model of curved plus extended groove incision.	41.39 × 24.40 × 32.84	0.00404158	0.00059486

### Analysis of numerical results of diversion

#### Straight model of spindle incision

**Figure 4:**
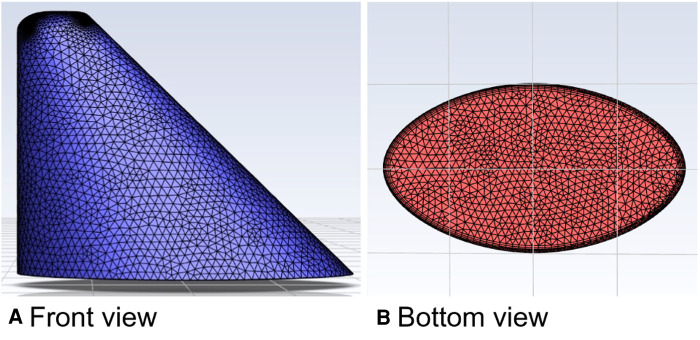
Straight model of the spindle incision.

The straight model of spindle incision was calculated using a coupled algorithm (Figure 4). The fluid was set as a non-Newtonian fluid; the Carreau model was used; the remaining material parameters were the same as water, and the density was 998.2 kg/m^3^. The velocity was increased by a factor of 100 at a calculated temperature of 27 °C, and the outlet flow rate was calculated in 200 s.

**Figure 5:**
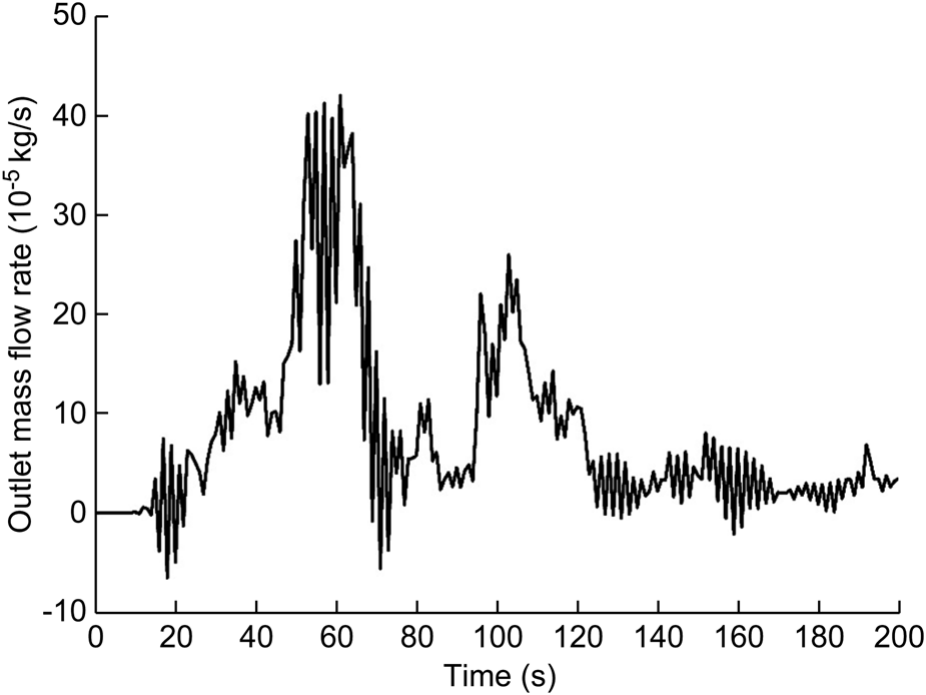
The outlet mass flow rate monitoring graph of the straight model of the spindle incision.

In this calculation example, the outlet mass flow rate was monitored, setting the number of steps to 200, step length to 1.0 s, and the physical time to 200 s. Figure 5 shows the outlet mass flow rate monitoring graph.

As seen in Figure 5, the mass flow rate gradually increased between 0 and 70 s, and the flow rate reached the maximum value of 4.2105510^−4^ kg/s at 61 s. After 70 s, the overall mass flow rate tended to slow until the curve flattened. Due to the viscous buildup effect of tissue fluid, the outlet mass flow curve was wavy, and the phenomenon of slight backflow occurred. Using the numerical integration method to integrate f(t), the total outlet flow *Q* of the straight model of spindle incision within 200 s was obtained.


(5) $$Q=\int_{0}^{200}\;f\;(t)dt\;=\;0.0157122684\;kg\;$$

#### Curved model of spindle incision

**Figure 6:**
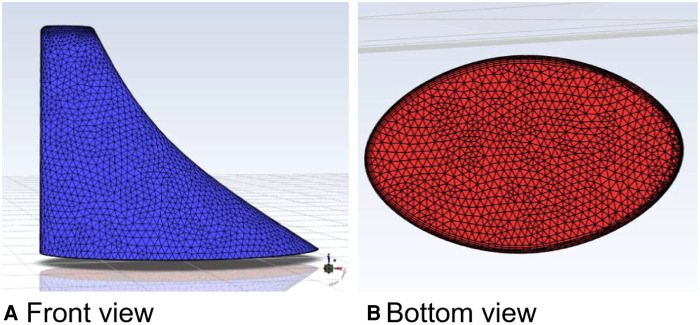
Curved model of the spindle incision.

The calculation algorithm and material parameters of the curved model of spindle incision (Figure 6) were the same as those of the straight model of spindle incision. The velocity was increased by a factor of 100 at a calculated temperature of 27 °C, and the outlet flow rate was calculated in 200 s.

**Figure 7:**
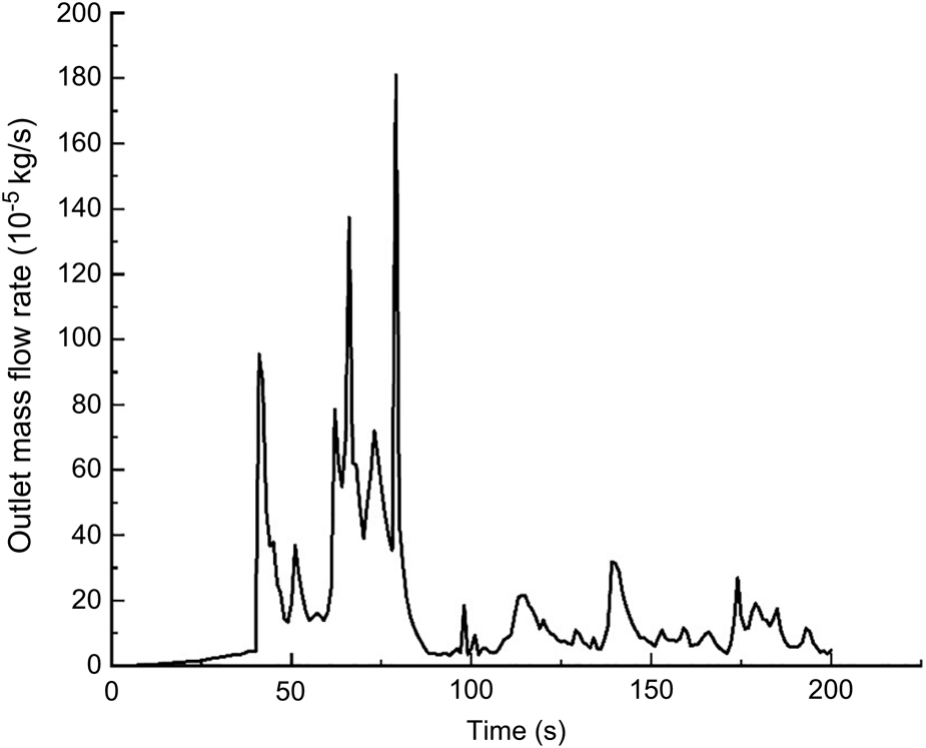
The outlet mass flow rate monitoring graph of the curved model of the spindle incision.

In this calculation example, the outlet mass flow rate was monitored, setting the number of steps to 200, step length to 1.0 s, and the physical time to 200 s. Figure 7 shows the outlet mass flow rate monitoring graph.

As seen in Figure 7, the mass flow rate gradually increased between 0 and 50 s. The flow rate reached the maximum value of 1.8010^−3^ kg/s by 79 s, and the overall mass flow rate showed an increasing trend between 50 s and100 s. While in the 100–200 s stage, the mass flow rate reached a relatively flat state. Using the numerical integration method to integrate f(t), the total outlet flow *Q* of the curved model of spindle incision within 200 s was obtained.


(6) $$Q=\int_{0}^{200}\;f\;(t)dt\;=\;0.03145174915\;kg\;$$

#### Straight model of curved incision

**Figure 8:**
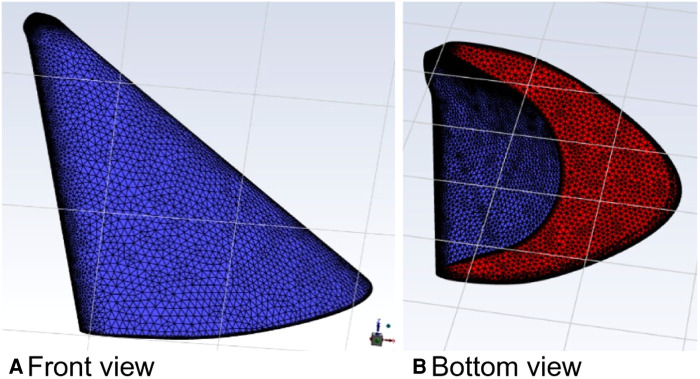
Straight model of the curved incision.

The straight model of curved incision (Figure 8) was also calculated using the coupled algorithm, and the remaining relevant parameters were the same as those of the spindle incision. The velocity was increased by a factor of 100 at a calculated temperature of 27 °C, and the outlet flow rate was calculated in 200 s.

**Figure 9:**
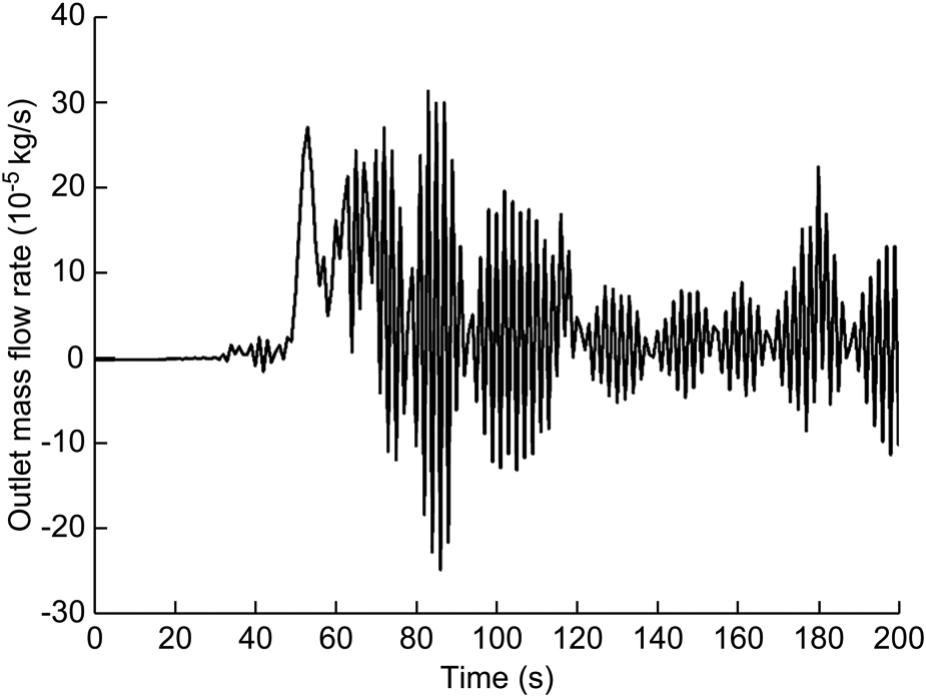
The outlet mass flow rate monitoring graph of the straight model of the curved incision.

In this calculation example, the outlet mass flow rate was monitored, setting the number of steps to 200, step length to 1.0 s, and the physical time to 200 s. Figure 9 shows the outlet mass flow rate monitoring graph.

As seen in Figure 9, the mass flow rate was relatively flat between 0 and 50 s. The flow rate reached a maximum value of 9.1610^−4 ^kg/s by 83 s. Between 100 and 200 s, the mass flow rate was wavy, and the phenomenon of backflow occurred due to the mucus effect. Using the numerical integration method to integrate f(t), the total outlet flow *Q* of the curved model of spindle incision within 200 s was obtained.


(7) $$Q=\int_{0}^{200}\;f\;(t)dt=\;0.0074599621\;kg$$

#### Curved model of curved incision

**Figure 10:**
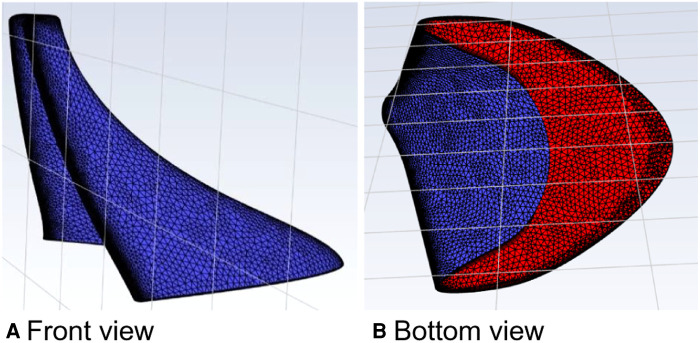
Curved model of the curved incision.

The parameters of the relevant properties of the curved model of curved incision (Figure 10) were consistent with the above calculation example. The velocity was increased by a factor of 100 at a calculated temperature of 27 °C, and the outlet flow rate was calculated in a physical time of 200 s.

**Figure 11:**
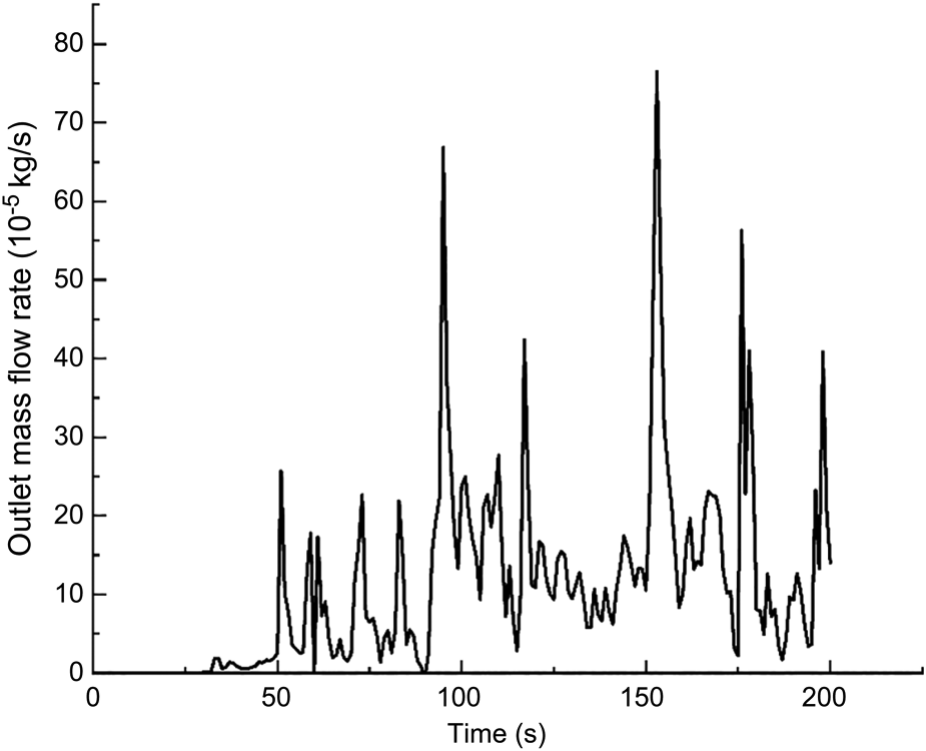
The outlet mass flow rate monitoring graph of the curved model of the curved incision.

In this calculation example, the outlet mass flow rate was monitored, setting the number of steps to 200, step length to 1.0 s, and the physical time to 200 s. Figure 11 shows the outlet mass flow rate monitoring graph.

As seen in Figure 11, the mass flow rate was relatively flat between 0 and 50 s. From 50 s to 100 s, the mass flow rate was wavy, and the backflow phenomenon occurred due to the mucus effect. The flow rate reached the maximum value of 7.6510^−4^ kg/s at 153 s, and the mass flow rate was wavy. Using the numerical integration method to integrate f(t), the total outlet flow *Q* of the curved model of spindle incision within 200 s was obtained.


(8) $$Q\;=\int_{0}^{200}\;f\;(t)dt=\;0.021512666602395\;kg$$

#### Straight model of curved plus extended groove incision

**Figure 12:**
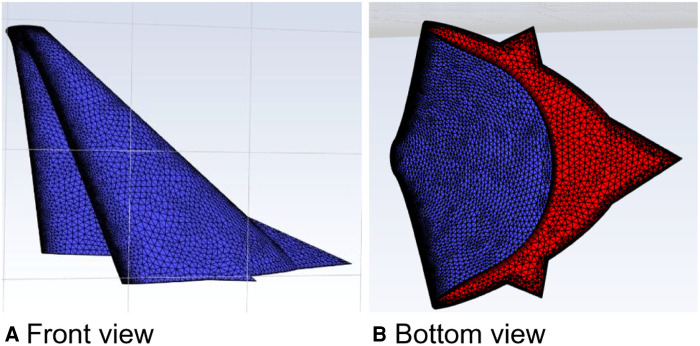
Straight model of the curved plus extended groove incision.

The property parameters of the straight model of curved plus extended groove incision (Figure 12) were consistent with the above calculation example, and the relevant modeling dimensions are shown in Table 6. The velocity was increased by a factor of 100 at a calculated temperature of 27 °C, and the outlet flow rate was calculated in a physical time of 200 s.

**Figure 13:**
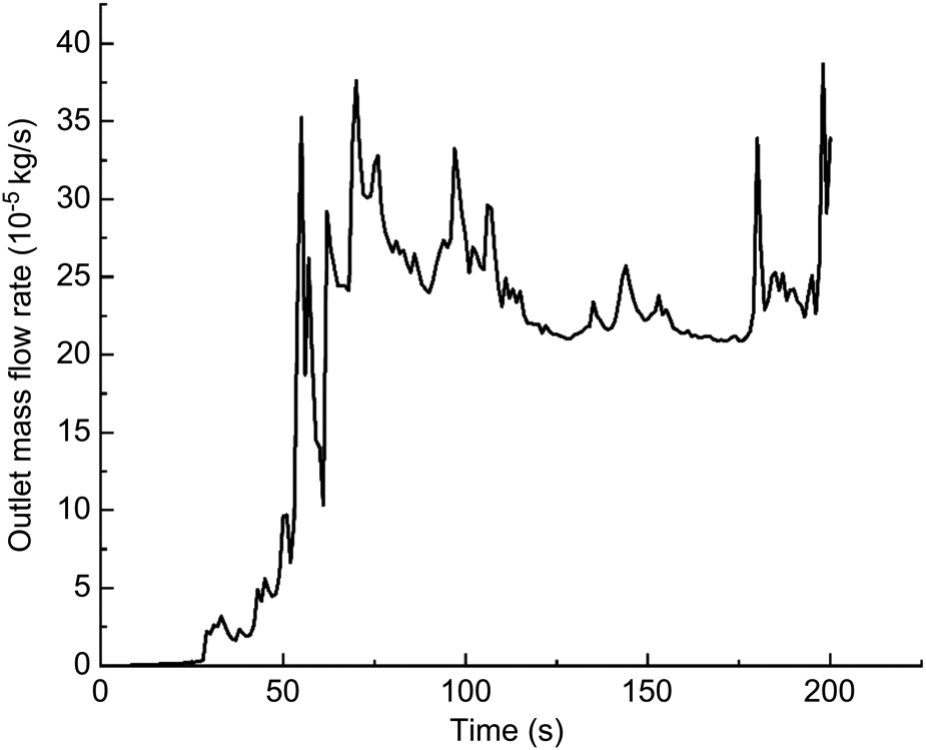
The outlet mass flow rate monitoring graph of the straight model of the curved plus extended groove incision.

In this calculation example, the outlet mass flow rate was monitored, setting the number of steps to 200, step length to 1.0 s, and the physical time to 200 s. Figure 13 shows the outlet mass flow rate monitoring graph.

As seen in Figure 13, the mass flow rate gradually increased between 0 and 50 s. The mass flow rate was wavy and relatively flat between 50 s and 100 s. The mass flow rate gradually increased from 150 to 200 s. The flow rate reached the maximum value of 3.8710^−4^ kg/s by 198 s. Using the numerical integration method to integrate f(t), the total outlet flow *Q* of the curved model of spindle incision within 200 s was obtained.


(9) $$Q\;=\int_{0}^{200}\;f\;(t)dt=\;0.036865853936\;kg$$

#### Curved model of curved plus extended groove incision

**Figure 14:**
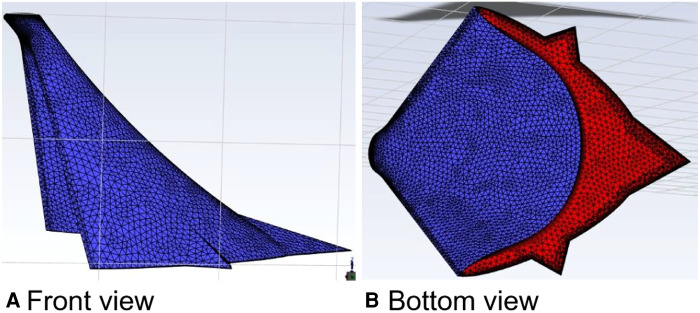
Curved model of the curved plus extended groove incision.

The parameters of the relevant properties of the curved model of curved plus extended groove incision (Figure 14) were the same as those of the above model. The velocity was increased by a factor of 100 at a calculated temperature of 27 °C, and the outlet flow rate was calculated in a physical time of 200 s.

**Figure 15:**
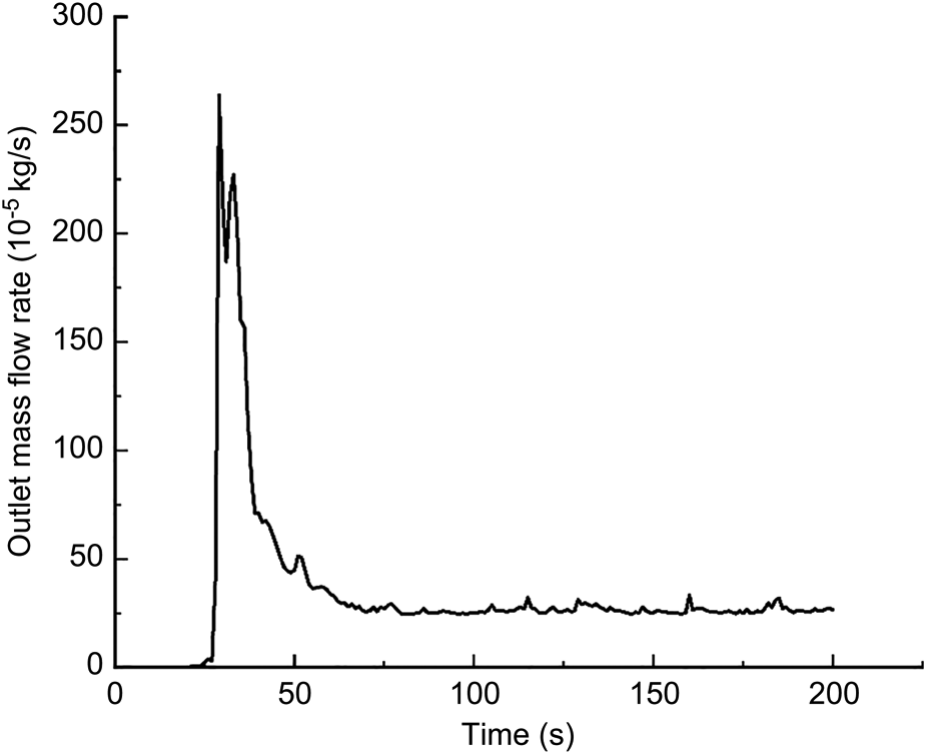
The outlet mass flow rate monitoring graph of the curved model of the curved plus extended groove incision.

In this calculation example, the outlet mass flow rate was monitored, setting the number of steps to 200, step length to 1.0 s, and the physical time to 200 s. Figure 15 shows the outlet mass flow rate monitoring graph.

As seen in Figure 15, the flow rate reached a maximum value of 2.6410^−3^ kg/s by 29 s. The mass flow rate was wavy and relatively flat between 50 s and 200 s. Using the numerical integration method to integrate f(t), the total outlet flow *Q* of the curved model of spindle incision within 200 s was obtained.


(10) $$Q\;=\int_{0}^{200}\;f\;(t)dt=\;0.066796601822\;kg$$

### Data interpretation

In this study, the drainage incisions with different shapes were modeled by SolidWorks, and the built models were imported into FLUENT finite element software. The drainage effects of different incision models were analyzed numerically, and the drainage flows of different incisions were calculated for a certain period of time. The following conclusions were drawn from this study:

1. After modeling with the same length, width, and depth, the areas of different incisions were as follows: straight model of spindle incision: 0.00085895 m^2^, straight model of curved incision: 0.0010334 m^2^, straight model of curved plus extended groove incision: 0.00059484 m^2^, curved model of spindle incision: 0.00085895 m^2^, curved model of curved incision: 0.00102991 m^2^, and curved model of curved plus extended groove incision: 0.00059486 m^2^. Thus, the area of the curved plus extended groove incision < the area of the spindle incision < the area of the curved incision (Table 6).
2. Using a mass flow inlet velocity 100 times the true flow velocity, a physical time of 200 s, and the same parameters set for each incision model, the total outlet flows of the different incision models were as follows: straight model of spindle incision: 0.0157122684 kg, straight model of curved incision: 0.0074599621 kg, straight model of curved plus extended groove incision: 0.036865853936 kg, curved model of spindle incision: 0.03145174915 kg, curved model of curved incision: 0.021512666602395 kg, and curved model of curved plus extended groove incision: 0.066796601822 kg. Therefore, in terms of the total outlet flow, that of the curved plus extended groove incision > that of spindle incision > that of curved incision, and the total outlet flow of curved incision > the total outlet flow of straight incision (Table 7).
3. The curved plus extended groove model had the smallest trauma area, but the drainage effect was better than that of the spindle and curved incisions, and the curved incision had better drainage effect than that of the straight incision.

**Table 7:** The outlet area and outlet flow of different incision models.

Model	The area (m^2^)	The total outlet flow (kg)
Straight model of spindle incision	0.00085895	0.0157122684
Curved model of spindle incision	0.00085895	0.03145174915
Straight model of curved incision	0.0010334	0.0074599621
Curved model of curved incision	0.00102991	0.021512666602395
Straight model of curved plus extended groove incision	0.00059484	0.036865853936
Curved model of curved plus extended groove incision.	0.00059486	0.066796601822

## Discussion

For the clinical management of high-grade complex anal fistulas with perianal pus accumulation, drainage of the abscess is an immediate priority (10) and a prerequisite for many pharmacological treatments (11). The proper incision design facilitates adequate drainage of pus and there is no damage to the sphincter muscle and no risk of anal incontinence (12).

In contrast to the traditional clinical practice, this paper uses incisional models for drainage simulation to guide the selection of incisions for high-grade complex anal fistulas through cross-disciplinary application. However, due to the diversity of clinical medical conditions and environments, many external factors cannot be taken into account when performing biomechanical modelling; therefore, the following shortcomings exist in this study: (1) when conducting a study on the effect of wound drainage, the default is that the amount of pus in the patient is not affected by perioperative medications such as antibiotics and is not affected by the patient's own constitution; (2) for the determination of drainage flow is by measuring the weight of gauze, which is a relatively low accuracy method. It is necessary to find a more reasonable measurement method in subsequent studies; (3) the research content of this paper is implemented based on the idealized incision model, which is weakly integrated with clinical practice, which is also the focus of subsequent studies.

Subject to the idealization of conditions in the corresponding areas, the results of this study showed that the curved plus extended groove incision had the smallest area and the best drainage compared to the curved and spindle incisions. In the authors' opinion, the curved plus extension groove incision removes less perianal skin and fatty tissue and does not damage the sphincter muscle, while ensuring the outer diameter of the incision and reducing the total area of the incision, which is in line with the current principle of minimally invasive surgery. The V-shaped expansion slot opens to form a more unobstructed drainage channel, which can help to heal the anal fistula with more adequate drainage of the postoperative incision, maintain the original shape of the anus, and greatly improve the patient's quality of life based on good protection of the anal shape. This study demonstrates that the drainage effect of the curved incision is better than that of the straight incision; thus, care should be taken to make a flared wound incision with narrow inner and outer width and curved edge, such that the drainage is more usual and the granulation tissue grows from the base to fill the wound gradually, avoiding pseudo-healing.

In conclusion, the curved plus extended groove incision is worth promoting for patients with high complex anal fistulas on account of minimal perianal tissue damage and good drainage.

## Data Availability

The original contributions presented in the study are included in the article/Supplementary Material, further inquiries can be directed to the corresponding author/s.
